# Visceral Obesity Predicts Fewer Lymph Node Metastases and Better Overall Survival in Colon Cancer

**DOI:** 10.1007/s11605-015-2834-z

**Published:** 2015-05-05

**Authors:** Se Woo Park, Hang Lak Lee, Eun Young Doo, Kang Nyeong Lee, Dae Won Jun, Oh Young Lee, Dong Soo Han, Byung Chul Yoon, Ho Soon Choi, Kang Hong Lee

**Affiliations:** Department of Internal Medicine, Institute of Gastroenterology, Hallym University College of Medicine, Hallym University Dongtan Sacred Heart Hospital, Gyeonggi-do, South Korea; Department of Internal Medicine, Institute of Gastroenterology, Hanyang University Hospital, Seoul, South Korea; Department of Internal Medicine, Healthcare Research Institute, Seoul National University Hospital Healthcare System Gangnam Center, Seoul, South Korea; Department of Surgery, Hanyang University Hospital, Seoul, South Korea

**Keywords:** Obesity, Visceral fat area, Lymph node, Metastasis, Colon cancer

## Abstract

**Background:**

The relationship between visceral obesity and colon cancer outcome has not been well studied. The goal of this study was to determine the impact of visceral obesity on lymph node (LN) metastasis and overall survival (OS) in colon cancer.

**Materials and Methods:**

Metastatic LN ratio (MLR) was defined as the number of involved nodes by tumor divided by the total number of resected LNs. Visceral (VFA) and subcutaneous fat areas (SFA) were determined by measuring abdominal fat volume distribution via CT scan, and visceral obesity was defined as a VFA to total fat area ratio (V/T) > 0.29.

**Results:**

In a multivariate analysis among 186 patients, there were inverse associations between V/T and MLR (OR = 0.413, 95 % CI = 0.216–0.789, *P* = 0.007). Furthermore, patients with visceral obesity tended to have significantly better OS than patients with non-visceral obesity.

**Conclusions:**

Higher V/T ratios which indicate referring to visceral obesity was significantly associated with decreased MLR and better OS for CRC.

## Introduction

Obesity is an even more prevalent issue in the world, but comparable data on associations with cancer are lacking.^1^ In recent years, obesity has been recognized as one of the possible non-surgical causes of postoperative adverse events and longer hospital stay after colorectal surgery.^2^ Another systemic review^3^ demonstrated that visceral obesity, especially, is associated with an increased risk of longer hospital stay, higher morbidity, and longer operative time after colon surgery. However, controversies exist regarding the correlation between visceral obesity and the outcome of colon cancer because of inconsistent results among the studies. One of the most important attributing factors for inconsistent results is the extent of lymph node (LN) metastasis that is a major determinant for the staging and prognosis of colon cancer and often guides therapeutic decisions. Especially, wide variations in number of metastatic LN among recovered LNs after colon resection exist according to the patient’s anatomy, the biological aggressiveness of the tumor, and the surgical techniques.^4^,^5^

In regard to colorectal surgery, visceral obesity contributes to the technical limitations and is a known predisposing factor for adverse events following surgery. In one largest cohort study,^6^ authors show that visceral obesity was associated with significantly more anastomotic leakage, pneumonia, wound infections and reoperations, and greater duration of hospital stay. Another study^7^ evaluated the importance of LN metastasis in colon cancer found that visceral obesity was associated with a lower likelihood of metastatic LN involvement because excess fat may limit accessibility to LNs located deep in the adipose tissue around major abdominal vessels not included in the routine en bloc resection.

Although the clinical significance of visceral obesity for LN involvement is well documented, little has been known about the real prognosis of colon cancer from visceral obesity not technical limitation such as inadequate LN dissection. Therefore, we investigated metastatic LN ratio (MLR), which is a powerful independent prognostic factor in colon cancer, even when only a few LN metastases are found,^8^ not simple number of LN involvement. MLR can show the quantity of metastasis to LNs; hence, advanced tumors must have higher range of MLR.

Because there are few studies that have investigated the effects of visceral obesity on LN metastasis, a retrospective study was performed to assess visceral obesity determined by computed tomography (subcutaneous fat area [SFA], visceral fat area [VFA], and visceral fat percentage) as a means of predicting LN metastasis and overall survival (OS) in a cohort of subjects with colon cancer.

## Materials and Methods

### Patients and Study Protocol

This retrospective study was initiated by reviewing the medical records of patients who underwent a surgical tumor resection due to histologically proven colon cancer in the Hanyang University Hospital Medical Center between 2003 and 2008. Detailed information was obtained from the computerized clinical information system, including demographic information, height and weight, laboratory findings, pathological findings, and semi-automated assessment of the subcutaneous and visceral fat compartments on multi-detector computed tomography (MDCT). A total of 278 patients with colon cancer underwent surgical tumor resection with regional lymphadenectomy, which is dissection of at least the group 1 LNs. In 186 patients with colon cancer, fat measurement was evaluated with preoperative MDCT and these patients were included in the study. Based on our exclusion criteria, patients with limited and palliative resections or those with emergency surgery for tumor-related complications (hemorrhage, ileus, perforation) were not considered for this subgroup. Furthermore, patients who were diagnosed with histologic subtypes other than adenocarcinoma (e.g., small cell carcinoma or neuroendocrine tumor) were excluded.

### Fat Measurement

BMI was calculated as weight divided by height squared. Patients were divided into four groups based on the National Institute of Health (NIH) classification for obesity: normal weight (18.5 ≤ BMI <25), underweight (BMI <18.5), overweight (25 ≤ BMI <30) and obese (30 ≤ BMI). Semi-automated assessment of subcutaneous and visceral fat compartments was performed using a dedicated software package (Fat Assessment Tool, EBW version 4.5, Philips Healthcare). The transverse cross section at the umbilical level was used to derive all abdominal fat measurements, as previously described and validated.^9^,^10^ The vendor-default histogram method was used, which determines the average attenuation value (in Hounsfield units) and standard deviation (SD) obtained from a range of −400 to 0 HU on a selected slice. Fat regions are then defined as the area enclosed under the fat histogram curve. Subcutaneous fat is defined as fat that is superficial to the abdominal wall musculature, whereas visceral fat is deep in the muscular wall and includes the mesenteric, subperitoneal, and retroperitoneal components. After the boundaries for subcutaneous and visceral compartments had been adjusted at the umbilical level, automated fat segmentation was performed, which can be further manipulated by the user to include or exclude focal regions if needed. The program then derives the subcutaneous fat area (SFA), the visceral fat area (VFA), total fat area (TFA = SFA + VFA), and the percentage of visceral fat to total fat area (V/T = VFA/TFA × 100). V/T was calculated to provide a single measure of abdominal fat, as published previously.^11^,^12^ Elevated V/T indicated higher visceral fat compared with subcutaneous fat, and a threshold was set at V/T = 29 % to define visceral obesity (V/T ≤ 29 % indicated subcutaneous obesity (VFs) and V/T > 29 % indicated visceral obesity (VFv)).^12^,^13^

### Clinicopathologic Data

Individual pathologic data were collected including maximum tumor diameter (mm), pathologic tumor stage (including T and N stage), tumor location, degree of tumor differentiation, and presence of lympho-vascular invasion or peri-neural invasion. In addition, the number of examined LNs, the number of metastatic LNs, and the metastatic LN ratio (MLR; number of metastatic LNs/number of examined LNs) was determined for each patient. LNs were recovered by routine mesenteric dissection by the pathologist and LN metastases were determined from subsequent pathologic reports. All pathologic results including MLR were interpreted by an independent pathologist who had no knowledge of the fat measurement data or clinical information. Furthermore, the pT and pN categories were based on the 2002 International Union Against Cancer and American Joint Committee on Cancer pTNM classification (i.e., pT category: pT1 [mucosa or submucosa infiltration] vs pT2 [muscularis propria] vs pT3 [subserosa or beyond without other organs involvement] vs pT4 [extension to other structures or perforates the visceral peritoneum], pN category: pN0 [no metastasis] vs pN1 [1 to 3 metastatic LNs] vs pN2 [4 or more metastatic LNs]).^14^ Right-sided colon cancers were defined as those arising from the cecum to the transverse colon. Left-sided colon cancers were defined as those arising from the splenic flexure down to and including the recto-sigmoid junction.^15^,^16^

Patient follow-up was conducted until death or the last contact date. Patient follow-up ended on June 30, 2010 and the mean follow-up was 45 months. During the follow-up period, 50 patients died of recurrence or metastasis, and survival time ranged from 4 to 89 months.

MLR is defined as the number of involved LNs divided by the number of dissected LNs. The MLR cutoff was designated at 18 %. Thus, MLR was categorized as MLR = 0 %, MLR < 18 % and MLR ≥ 18 %.^17^,^18^ Adjuvant therapy was administered according to pathologic stage and physician recommendation.

### Statistical Analysis

Descriptive statistics were provided for binary and continuous variables using incidence frequency (%) and mean ± standard deviation. The chi-square test was used to compare binary variables, and two-sample *t* test was used to compare continuous variables. Multivariate logistic regression analysis adjusted by confounding factors was used to assess inter-group differences in MLR. The Kaplan-Meier model with log-rank test was used to assess the impact of different characteristics on survival. Hazard ratios were calculated using the Cox proportional hazard regression model to assess potential predictors of survival. All statistical analyses were performed using SPSS software for windows, version 17 (SPSS Inc. Chicago, IL, USA).

### Ethics Statement

The study described in this report was approved by the Ethics Committee of Hanyang University School of Medicine, Seoul, Korea, in accordance with the Declaration of Helsinki.

## Results

### Patient Characteristics

**Fig. 1:**
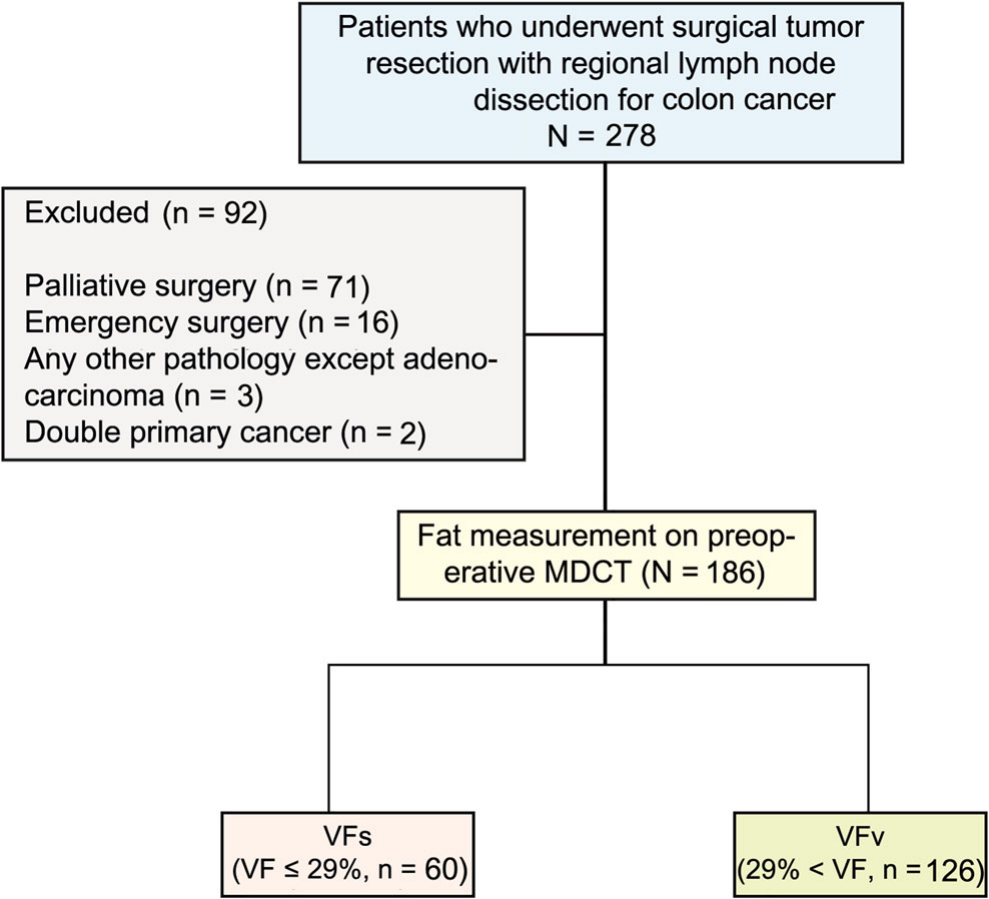
Flow of patients throughout the study. Medical records were reviewed for 278 consecutive inpatients, all of whom underwent surgical tumor resection with regional lymphadenectomy (dissection of at least the group 1 lymph nodes). In 186 patients with colon cancer with radical resection, fat measurement was evaluated by preoperative MDCT. A total of 92 patients were excluded for the following reasons: palliative surgery (*n* = 71), emergency surgery for tumor-related complications (hemorrhage, ileus, perforation) (*n* = 16), any other pathology except adenocarcinoma (*n* = 3), or double primary cancer (*n* = 2)

Medical records were reviewed for 278 consecutive inpatients (Fig. 1), all of whom underwent surgical tumor resection with regional lymphadenectomy (dissection of at least the group 1 LNs). In 186 patients with colon cancer with radical resection, fat measurement was evaluated by preoperative MDCT. A total of 92 patients were excluded for the following reasons: palliative surgery (*n* = 71), emergency surgery for tumor-related complications (hemorrhage, ileus, perforation) (*n* = 16), any other pathology except adenocarcinoma (*n* = 3), or double primary cancer (*n* = 2).

Patients were divided into two groups: VFs (V/T ≤ 29 %, *n* = 60) and VFv (V/T > 29 %, *n* = 126). Table 1 shows the demographic and clinicopathologic characteristics of these two groups. The mean age of individuals in group VFs was 68.12 ± 9.16 years old, while the mean in group VFv was 65.43 ± 11.67 years old. There were slightly more males in both groups, although this was not significant (60.0 % in group VFs and 58.7 % in group VFv, *P* = 0.875). Mean BMI in the VFv group was significantly higher than in the VFs group (22.72 ± 3.05 vs. 24.21 ± 3.34, *P* = 0.004).

**Table 1:** Baseline characteristics according to abdominal obesity classification

Variables	VFs (*n* = 060)	VFv (*n* = 0126)	*P*
Age	68.12 ± 9.16	65.43 ± 11.67	0.090
Sex (male)	36 (60.0 %)	74 (58.7 %)	0.875
Body mass index (kg/m^2^)	22.72 ± 3.05	24.21 ± 3.34	0.004
Laboratory findings			
HbA1c	6.20 ± 0.63	6.12 ± 1.09	0.595
Total cholesterol	177.58 ± 41.28	173.48 ± 34.90	0.507
Maximum tumor diameter (mm)	20.12 ± 8.27	19.70 ± 7.47	0.731
pT stage			
T1	5 (8.3 %)	9 (7.1 %)	0.990
T2	5 (8.3 %)	11 (8.7 %)	
T3	44 (73.3 %)	94 (74.6 %)	
T4	6 (10.0 %)	12 (9.5 %)	
Tumor location			
Right side	22 (36.7 %)	49 (38.9 %)	0.872
Left side	38 (63.3 %)	77 (61.1 %)	
Histologic grade			
Well differentiated	2 (3.3 %)	10 (7.9 %)	0.114
Moderate differentiated	57 (95.0 %)	106 (84.1 %)	
Poorly differentiated	1 (1.7 %)	10 (7.9 %)	
Lymphatic invasion			
Negative	17 (28.3 %)	29 (23.0 %)	0.469
Positive	43 (71.7 %)	97 (77.0 %)	
Vascular invasion			
Negative	56 (93.3 %)	108 (85.7 %)	0.152
Positive	4 (6.7 %)	18 (14.3 %)	
Peri-neural invasion			
Negative	34 (56.7 %)	73 (57.9 %)	0.875
Positive	26 (43.3 %)	53 (42.1 %)	
No. of retrieved LN	26.08 ± 10.27	28.07 ± 15.37	0.298
MLR			
MLR = 0 %	14 (23.3 %)	55 (43.7 %)	<0.001
MLR < 18 %	12 (20.0 %)	50 (39.7 %)	
18 % ≤ MLR	34 (56.7 %)	21 (16.7 %)	
Adjuvant CTX	27 (45.0 %)	68 (54.0 %)	0.275

Data were presented as [*n* (%)] and comparisons were made with chi-square. Data were presented as (mean ± SD) and comparison was made with Student’s *t* test

*VFs* subcutaneous obesity as V/T ≤ 29 %, *VFv* visceral obesity as V/T > 29 %, *pT stage* pathologic T stage, *CTX* chemotherapy, *RCC* right-sided colon cancers arising from the cecum to the transverse colon, LCC left-sided colon cancers arising from the splenic flexure down to and including the rectosigmoid junction, *MLR* metastatic lymph node ratio which is categorized as MLR = 0 %, MLR < 18 %, and 18 % ≤ MLR

There were no significant differences between the two groups in terms of the proportion of tumor location (*P* = 0.872) or maximum tumor diameter (20.12 ± 8.27 vs 19.70 ± 7.47, respectively; *P* = 0.731). Additionally, the proportion of pathologic T stage and histologic grade were not statistically different between the groups. Other pathologic variables including lympho-vascular invasion and peri-neural invasion were similar between the two groups of patients. Furthermore, although the proportion of MLR was significantly different between both groups (*P* < 0.001), the mean numbers of retrieved LNs were not statistically different between the groups (26.08 ± 10.27 vs 28.07 ± 15.37, respectively; *P* = 0.298). There was no significant difference between the two groups in terms of adjuvant chemotherapy after curative surgery.

### Factors Associated with LN Metastasis

Univariate and multivariate logistic regression analysis was performed to discern the contribution of variable factors on the LN metastasis, which was categorized as a “node negative” or “node positive.” Potentially associated variables for LN metastasis are presented in Table 2. When LN metastasis was the dependent variable in univariate logistic regression analysis, the following factors were significant: peri-neural invasion presence (*P* = 0.002) and lower V/T ratio (*P* = 0.008). In multivariate analysis, LN metastasis was significantly associated with peri-neural invasion presence (hazard ratio [HR] = 3.621, 95 % confidence interval [CI] = 1.686–7.777, *P* = 0.001) and lower V/T ratio (for higher V/T ratio; HR = 0.291, 95 % CI = 0.133–0.638, *P* = 0.002).

**Table 2:** Univariate and multivariate regression analysis on the clinicopathologic factors in node-negative and node-positive groups

Factors	Node negative (*n* = 69)	Node positive (*n* = 117)	Univariate, hazard ratio (95 % CI)	*P*	Multivariate, hazard ratio (95 % CI)	*P*
Age						
≤60	13 (18.8)	31 (26.5)	1		1	
>60	56 (81.2)	86 (73.5)	0.644 (0.310–1.336)	0.237	0.560 (0.238–1.316)	0.184
Sex						
Male	40 (58.0)	70 (59.8)	1		1	
Female	29 (42.0)	47 (40.2)	0.850 (0.583–1.241)	0.401	0.926 (0.506–1.695)	0.803
BMI						
Normal	44 (63.8)	79 (67.5)	1		1	
Underweight	3 (4.3)	6 (5.1)	1.114 (0.265–4.674)	0.883	1.041 (0.214–5.050)	0.961
Overweight	21 (30.4)	27 (23.1)	0.716 (0.363–1.412)	0.825	0.916 (0.421–1.991)	0.825
Obese	1 (1.4)	5 (4.3)	2.785 (0.315–24.597)	0.357	6.765 (0.662–69.109)	0.107
Tumor location						
Rt side colon	24 (34.8)	47 (40.2)	1		1	
Lt side colon	45 (65.2)	70 (59.8)	0.794 (0.428–1.474)	0.465	0.884 (0.434–1.793)	0.732
pT stage						
T1	6 (8.7)	8 (6.8)	1		1	
T2	6 (8.7)	10 (8.5)	1.250 (0.289–5.407)	0.765	1.862 (0.336–10.308)	0.477
T3	52 (75.4)	86 (73.5)	1.240 (0.408–3.775)	0.704	1.167 (0.272–5.009)	0.835
T4	5 (7.2)	13 (11.1)	1.950 (0.445–8.548)	0.376	1.671 (0.267–10.455)	0.583
Histology						
Well diff	3 (4.3)	9 (7.7)	1		1	
Moderate diff	65 (94.2)	98 (83.8)	0.503 (0.131–1.926)	0.098	0.273 (0.059–1.272)	0.098
Poorly diff	1 (1.4)	10 (8.5)	3.333 (0.292–38.082)	0.333	2.623 (0.184–37.469)	0.477
Lymphatic inv						
Absent	19 (27.5)	27 (23.1)	1		1	
Present	50 (72.5)	90 (76.9)	1.267 (0.641–2.503)	0.496	1.084 (0.435–2.701)	0.862
Vascular inv						
Absent	60 (87.0)	104 (88.9)	1		1	
Present	9 (13.0)	13 (11.1)	0.833 (0.336–2.065)	0.694	0.582 (0.186–1.820)	0.352
PNI						
Absent	50 (72.5)	57 (48.7)	1		1	
Present	19 (27.5)	60 (51.3)	2.770 (1.460–5.257)	0.002	3.621 (1.686–7.777)	0.001
V/T^a^						
≤29 %	14 (20.3)	46 (39.3)	1		1	
>29 %	55 (79.7)	71 (60.7)	0.393 (0.196–0.787)	0.008	0.291 (0.133–0.638)	0.002

“Node negative” is defined as group that no metastatic lymph nodes were retrieved in. “Node positive” is defined as group that at least one or more metastatic lymph nodes were retrieved in

*BMI* body mass index, *Rt* right, *Lt* left, *pT stage* pathologic T stage, *diff* differentiated, *inv* invasion, *PNI* perineural invasion, *V*/*T* the percentage of visceral fat to total fat area

^a^Elevated V/T indicated higher visceral fat compared with subcutaneous fat, and a threshold was set at V/T = 29 % to define visceral obesity (V/T ≤ 29 % indicated subcutaneous obesity and V/T > 29 % indicated visceral obesity)

### Factors Associated with Metastatic LNs Ratio in Node-Positive Patients

In node-positive patients, univariate and multivariate logistic regression analysis which was categorized as a “MLR < 18 %” or “MLR ≥ 18 %” showed that the variables potentially influencing MLR were presented in Table 3. When MLR was the dependent variable in multivariate analysis, MLR was significantly associated only with lower V/T ratio (for higher V/T ratio; HR = 0.111, 95 % CI = 0.040–0.307, *P* < 0.001). Furthermore, there was a marginal significance regarding with tumor location (for left side colon cancer; HR = 2.531, 95 % CI = 0.963–6.653, *P* = 0.060).

**Table 3:** Univariate and multivariate regression analysis on the clinicopathologic factors for metastatic lymph node ratio in node-positive patients

Factors	MLR < 18 % (*n* = 62)	MLR ≥ 18 % (*n* = 55)	Univariate, hazard ratio (95 % CI)	*P*	Multivariate, hazard ratio (95 % CI)	*P*
Age						
≤60	15 (24.2)	16 (29.1)	1		1	
>60	47 (75.8)	39 (70.9)	0.778 (0.342–1.771)	0.550	0.561 (0.194–1.623)	0.286
Sex						
Male	36 (58.1)	34 (61.8)	1		1	
Female	26 (41.9)	21 (38.2)	0.855 (0.407–1.796)	0.679	0.972 (0.380–2.488)	0.954
BMI						
Normal	39 (62.9)	40 (72.7)	1		1	
Underweight	2 (3.2)	4 (7.3)	1.950 (0.338–11.264)	0.883	1.407 (0.132–15.061)	0.778
Overweight	16 (25.8)	11 (20.0)	0.670 (0.277–1.625)	0.825	0.693 (0.229–2.096)	0.516
Obese	5 (8.1)	0				
Tumor location						
Rt side colon	29 (46.8)	18 (32.7)	1		1	
Lt side colon	33 (53.2)	37 (67.3)	1.806 (0.851–3.833)	0.465	2.531 (0.963–6.653)	0.060
pT stage						
T1	3 (4.8)	5 (9.1)	1		1	
T2	7 (11.3)	3 (5.5)	0.257 (0.036–1.843)	0.176	0.494 (0.044–5.497)	0.566
T3	47 (75.8)	39 (70.9)	0.498 (0.112–2.216)	0.360	1.969 (0.216–17.946)	0.548
T4	5 (8.1)	8 (14.5)	0.960 (0.156–5.900)	0.965	8.671 (0.540–139.347)	0.127
Histology						
Well diff	4 (6.5)	5 (9.1)	1		1	
Moderate diff	52 (83.9)	46 (83.6)	0.708 (0.179–2.794)	0.622	0.327 (0.059–1.810)	0.200
Poorly diff	6 (9.7)	4 (7.3)	0.533 (0.086–3.307)	0.500	0.549 (0.057–5.257)	0.603
Lymphatic inv						
Absent	12 (19.4)	15 (27.3)	1		1	
Present	50 (80.6)	40 (72.7)	0.640 (0.269–1.521)	0.312	0.324 (0.074–1.413)	0.134
Vascular inv						
Absent	53 (85.5)	51 (92.7)	1		1	
Present	9 (14.5)	4 (7.3)	0.462 (0.134–1.594)	0.222	0.253 (0.040–1.593)	0.143
PNI						
Absent	29 (46.8)	28 (50.9)	1		1	
Present	33 (53.2)	27 (49.1)	0.847 (0.410–1.753)	0.655	1.517 (0.521–4.416)	0.445
V/T^a^						
≤29 %	12 (19.4)	34 (61.8)	1		1	
>29 %	50 (80.6)	21 (38.2)	0.148 (0.064–0.341)	<0.001	0.111 (0.040–0.307)	<0.001

*BMI* body mass index, *Rt* right, *Lt* left, *pT stage* pathologic T stage, *diff* differentiated, *inv* invasion, *PNI* perineural invasion, *V*/*T* the percentage of visceral fat to total fat area, *MLR* metastatic lymph node ratio which was defined as the number of involved nodes by tumor divided to the total number of resected lymph nodes and categorized as MLR < 18 % and 18 % ≤ MLR

^a^Elevated V/T indicated higher visceral fat compared with subcutaneous fat, and a threshold was set at V/T = 29 % to define visceral obesity (V/T ≤ 29 % indicated subcutaneous obesity and V/T > 29 % indicated visceral obesity)

### Survival Analysis

**Fig. 2:**
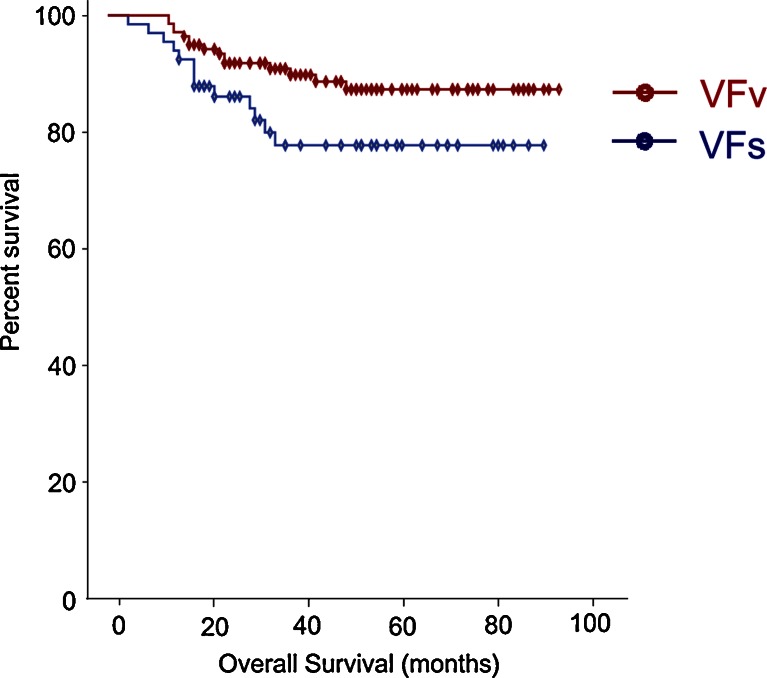
Survival curves in patients according to abdominal obesity classification. Kaplan-Meier model estimates of survival probability according to V/T classification for the 186 study participants. Mean OS was 69.71 ± 3.97 months in the VFs subgroup vs 80.02 ± 2.17 months in the VFv subgroup. Patients with visceral obesity tended to have better OS than non-visceral obesity patients with marginal significance (log-rank test: V/T ≤ 29 % (VFs) vs 29 % < V/T (VFv), *P* = 0.057) (*red line*, VFv; *blue line*, VFs)

A Kaplan-Meier model was used to estimate survival probability of OS stratified by V/T classification for the 186 study participants. Median OS had not been reached for any of the subgroups at the end of follow-up. Mean OS was 69.71 ± 3.97 months in the VFs subgroup vs 80.02 ± 2.17 months in the VFv subgroup. Patients with visceral obesity tended to have better OS than non-visceral obesity patients with marginal significance (*P* = 0.057) (Fig. 2).

## Discussion

This is the first report to use multiple quantitative visceral fat measurements (SFA, VFA, and TFA) to examine the associations between visceral obesity and LN metastasis or OS in patients with curative resection for colon cancer. The primary findings showed significant positive associations between MLR of colon cancer and peri-neural invasion. Also, there was a significant inverse association between MLR and visceral obesity. Many recent reports on colon cancer demonstrated that obese patients generally have lower chances of survival and more aggressive biological tumor features. One systematic review and meta-analysis^3^ of 1230 patients among nine studies reported that visceral obesity leads to a longer hospital stay, higher morbidity, and longer operative time after elective colon surgery. Furthermore, four of nine studies reported that LN retrieval was significantly lower in the visceral obese patients and overall outcome including survival was poorer than non-obese patients because of incomplete LN dissection.

Interestingly, our results demonstrated that BMI was not associated with overall outcome of colon cancer including LN metastasis or OS. In clinical practice, many studies have been used anthropometric index such as BMI, WC, or waist-to-hip ratio as surrogates of visceral obesity.^19^,^20^ However, according to one study, authors demonstrated that BMI is not an accurate index to quantify intra-abdominal visceral fat because of the different fat tissue distribution between individuals and various ethnic groups.^21^ Furthermore, one study^22^ tested the correlation between BMI and visceral obesity concluded that there was a weak positive correlation between BMI and VFA and no correlation between BMI and the VFA/SFA ratio. These results indicate that BMI alone is not an accurate index of visceral obesity. This suggests that VFA/SFA ratio is a more accurate index of visceral obesity than VFA alone or other anthropometric indices, as the latter also depends on patient size and not exclusively on distribution of adipose tissue. Interestingly, in colon cancer patients in this study, 67.7 % met the criteria for visceral obesity by V/T and only 3.2 % of patients had a BMI ≥ 30.

A previous study^7^ evaluated the importance of LN metastasis as a determinant of colon cancer patient prognosis and found that being overweight was associated with a lower likelihood of metastatic LN involvement. In a large intergroup trial (INT-0089), incomplete lymphadenectomy of various extents was attributed to limitation accessibility to LN located deep in the excess fat tissue around major vessels.^23^ This study emphasized the importance of the stage migration phenomenon and poorer prognoses related to inadequate LN dissection. In addition, if a patient was classified as node-negative, survival was also affected positively with an increase in the number of nodes analyzed; this observation has important clinical implications for surgeons operating on obese individuals. There is some debate over the number of nodes that must be examined to yield a reliable assessment of patient nodal status. In 1990, the Working Party Report to the World Congresses of Gastroenterology reviewed this topic and came to a consensus recommendation that at least 12 nodes must be sampled to adequately stage a patient.^24^ However, a study by Goldstein et al.^25^ found that the number of node-positive patients continued to increase until 17–20 nodes had been examined, leading to the conclusion that a minimum of 17 nodes should be analyzed. Similarly, Wong et al.^26^ found that significantly fewer nodes were examined in node-negative patients than in node-positive patients (14 vs 20 nodes), and to achieve a nodal positivity rate commensurate with the National Cancer Data Bank, at least 14 nodes should be examined. It is clear that to accurately stage a patient with colon cancer, it is best to evaluate as many nodes as possible. The number of LNs dissected in the current study ranged from 12 to 90, with a mean of 27.4 (26.08 ± 10.27 in VFs group vs 28.07 ± 15.37 in VFv group, *P* = 0.298). No patient had fewer than the required 12 nodes dissected.

However, in terms of statistics, the number of metastatic LNs increases according to the number of dissected nodes, suggesting that the pathologic N stage can be influenced by the extent of lymphadenectomy. In addition, the pathologic N stage may be changed by adding or reducing one positive LN, suggesting that colon cancer classified as N1 after limited LN dissection may be classified as N2 after extensive lymphadenectomy. Therefore, Chen et al.^27^compared the prognostic values of the MLR categories with that of the pathologic N (pN) categories in patients with no less than 12 LNs retrieved. Multivariate analysis showed that both MLR and LN involvement were independent prognostic factors. They proposed that MLR categories had better prognostic value than pN categories because the MLR categories had a higher hazard ratio than the pN categories. Another study by Schumacher et al.^17^compared the pathologic N stage with a classification based on MLR in patients who underwent curative tumor resection and determined that MLR is the most important prognostic factor for colon cancer. Based on this theoretical background, the present study used MLR as a more accurate prognostic value compared to traditional classification of LN metastasis in the TNM staging system.

In a Kaplan-Meier model with log-rank test between VFs group and VFv group, no differences were observed in overall survival rate, although MLR was significantly lower among patients with visceral obesity (VFv group). This lack of survival benefits might be related to differences in various confounding factors (e.g., regimen of the postoperative adjuvant chemotherapy or any other patient-related factors).

The limitations of this study include its retrospective design, which is subject to incomplete data and potential selection bias. Moreover, the use of visceral obesity as a risk stratification tool has its own disadvantages. First, an accepted V/T threshold for defining visceral obesity is currently lacking. Furthermore, accurate measurement of visceral obesity relies on CT imaging, which is expensive and impractical in everyday clinical practice. Other confounding factors that were not considered may have had an effect on the LN metastasis and OS. Especially, the lack of survival benefits observed in this study could be attributed to the confounding factors that were not considered such as adjuvant chemotherapy or adjusting for many baseline confounding factors that may dilute a potential survival benefit. Finally, this study did not analyze the disease-free survival rate or the loco-regional recurrence rate, for which LN metastasis is a very important independent risk factor in patients with colon cancer after curative resection.

## Conclusion

We found that a higher ratio of visceral fat was associated with a decreased LN metastasis or MLR, although there was no association between visceral obesity and overall survival for colon cancer. This was labeled the “buffering effect” and the underlying mechanism is still unknown. Further studies about MLR and local or systemic recurrence rates are warranted to clarify the association between survival rates and visceral fat distribution in colon cancer.
